# A Case Report of Meningitis due to *Borrelia* Species in Arizona

**DOI:** 10.5811/cpcem.61989

**Published:** 2026-09-26

**Authors:** Scott Austin, Tamlyn Tarzia, Griffith Saunders

**Affiliations:** Valleywise Health Medical Center, Emergency and Trauma Services, Phoenix, Arizona

**Keywords:** infectious disease, neurology, critical care

## Abstract

**Introduction:**

Lyme disease is most prevalent in the Northeast and Midwest regions of the United States, as well as northern California; however, cases have been reported in nonendemic areas, including the Southwestern United States. Atypical presentations or underreporting can cause delay in diagnosis.

**Case Report:**

We present the case of a 22-year-old previously healthy man, who presented to our emergency department in metropolitan Phoenix with five days of headache, fever, meningismus, and photophobia, and later developed focal neurologic deficits consistent with Lyme meningitis. Cerebrospinal fluid was notable for lymphocytic pleocytosis, and Western blot for *Borrelia burgdorferi* Immunoglobulin-M antibodies was positive, which led us to Lyme meningitis as the unifying diagnosis. He was treated with intravenous ceftriaxone and his symptoms resolved.

**Conclusion:**

This case highlights the importance of considering this pathology in nonendemic areas.

## INTRODUCTION

Lyme disease is caused by *Borrelia burgdorferi* and is the most prevalent vector-borne illness in the United States. The highest incidence is in the Northeast, Midwest, and northern Pacific Coast. However, Lyme disease. has been increasingly detected in areas that have traditionally been considered low risk, including the Southwest, as well as states neighboring high-endemic areas such as Ohio, Indiana, Iowa, and North Carolina.^5^

Lyme meningitis is a rare but serious manifestation of *Borrelia* infection. It typically manifests with headache and photophobia, although cranial neuritis and radiculoneuritis are also seen. It has been well-documented in the Northeast and Midwest but infrequently in other regions. The Wilderness Medical Society confirms that while *Borrelia* infections are rare in the Southwest, increasing travel patterns, climate change, and the spread of the vector species, may be contributing to an expanded geographic range of this pathology.^1^ Additionally, other *Borrelia* species such as *Borrelia mayonii* have been implicated in neurologic pathologies across the US, further suggesting that the prevalence of *Borrelia* infections outside endemic areas may be underreported.^3^ Continued surveillance is crucial to understanding the epidemiology of Lyme disease and its neurological sequelae in nontraditional areas.

## CASE REPORT

A 22-year-old man presented to two separate emergency departments (ED) in the urban metropolitan–Phoenix area with complaints of headache, photophobia, and fever. He was a professional boxer and had sustained a strike to the left parietal-occipital cranium on the day prior to index presentation. Noncontrast computed tomography of the head was negative for acute pathology or evidence of trauma. The patient was thought to have postconcussive headache and was discharged without further workup on both occasions. On the fifth day of symptoms and four days after the initial ED visit, the patient presented to our ED. He complained of persistent symptoms associated with meningismus and vomiting.

His initial vital signs were notable only for a temperature of 38.8 °C. On examination, he had decreased passive flexion of the neck secondary to pain but was without focal neurologic deficits. There was no evidence of dermatologic abnormalities or tick bites. Complete blood count revealed leukocytosis of 11.3 cells/mL (reference range, 3.4 cells/mL–11.0 cells/mL) with a neutrophilic predominance. Complete metabolic panel was notable for sodium of 126 mmol/L (137 mmol/L–145 mmol/L) and lactic acid of 2.9 mmol/L (0.7mmol/L-1.9 mmol/L). As there was clinical concern for meningitis, the patient underwent lumbar puncture. Initial cerebrospinal fluid analysis revealed increased protein and a pleocytosis with lymphocytic predominance.

The patient was admitted to the medicine floor and initiated on empiric antimicrobials for bacterial meningitis, which included ceftriaxone and vancomycin. Because *Varicella Zoster* and *Listeria* were deemed unlikely, coverage for those were not included. Further cerebrospinal fluid analysis was negative for bacterial and fungal cultures, herpes simplex virus types 1 and 2, parechovirus *and* enterovirus, West Nile virus antibodies, and *Coccidioides* antigen and antibodies. Antimicrobials were discontinued, but the patient continued to sustain fevers. On hospital day 6, he developed a left-sided facial droop and left-sided extremity weakness. His level of consciousness declined, and he became oriented only to self. Because there was concern for his ability to protect his airway, he was intubated and transferred to the medical intensive care unit.

Contrast-enhanced magnetic resonance imaging of the brain was unremarkable, but imaging of the cervical spine was notable for meningeal enhancement. Additional serologic studies revealed a positive Lyme antibody, and confirmatory *Borrelia Burgdorferi* immunoglobulin-M by Western blot was positive. Repeat lumbar puncture was negative for *Borrelia* species by polymerase chain reaction, although these results were thought to be affected by preceding antibiotics administration. Further history from the patient’s family revealed that that he had frequented mountainous areas in northern Arizona within three months prior to his presentation and was known to interact with wildlife. He had no other travel history. The diagnosis was most consistent with Lyme meningitis, and he subsequently received a 14-day course of intravenous ceftriaxone. His mental status improved, and neurologic deficits resolved.

## DISCUSSION

We describe the case of a young man who presented to the ED with headache and fever and later developed facial nerve palsy with unilateral weakness, thought to be secondary to Lyme meningitis in a nonendemic area. Guidelines from the Infectious Diseases Society of America and the American Academy of Neurology describe Lyme meningitis as a known early manifestation of disseminated *Borrelia burgdorferi* infection within the first months of exposure. Although Lyme disease is most prevalent in the Northeast, Midwest, and northern California, the Wilderness Medical Society notes that cases have been reported in nonendemic areas, including the southwestern US.

*CPC-EM Capsule*

What do we already know about this clinical entity?

*The literature regarding Borrelia infections, including the development of Lyme meningitis, is derived from endemic areas.*

What makes this presentation of disease reportable?

*This case highlights the expansion of Lyme disease into nonendemic areas, including the Southwestern United States.*

What is the major learning point?

*Clinicians should have a high index of clinical suspicion, even in nonendemic areas, as it changes management of these patients.*

How might this improve emergency medicine practice?

*The species differential diagnosis for bacterial meningitis could impact the serologies that are initially sent with cerebrospinal fluid samples and lead to earlier diagnosis.*

## CONCLUSION

Awareness of *Borrelia* infection outside endemic regions is critical to avoid missed or delayed diagnosis, given that the geographic range of vectors continues to expand. Emergency physicians in the Southwest should maintain a high index of suspicion for Lyme meningitis in patients with compatible neurologic complaints, particularly when the patient’s travel history is indeterminate. Prompt recognition and initiation of intravenous ceftriaxone (2 g daily for 10–28 days) is associated with favorable outcomes.
